# Factors Associated With Severity of Delirium Complicating COVID-19 in Intensive Care Units

**DOI:** 10.3389/fneur.2022.774953

**Published:** 2022-03-24

**Authors:** Domenico Madonna, Paolo Enrico, Valentina Ciappolino, Andrea Boscutti, Elisa Colombo, Nunzio Turtulici, Filippo Cantù, Guido Cereda, Giuseppe Delvecchio, Stefano De Falco, Monica Chierichetti, Monica Savioli, Giacomo Grasselli, Paolo Brambilla

**Affiliations:** ^1^Department of Neurosciences and Mental Health, Fondazione Istituto di Ricovero e Cura a Carattere Scientifico (IRCCS) Ca' Granda Ospedale Maggiore Policlinico, Milan, Italy; ^2^Department of Pathophysiology and Transplantation, University of Milan, Milan, Italy; ^3^Department of Anesthesia, Intensive Care and Emergency, Fondazione Istituto di Ricovero e Cura a Carattere Scientifico (IRCCS) Ca' Granda-Ospedale Maggiore Policlinico, Milan, Italy

**Keywords:** COVID-19, delirium, ARDS, prognosis, ICUs, psychiatric symptoms, cognition

## Abstract

The clinical outcome of the disease provoked by the SARS-CoV-2 infection, COVID-19, is largely due to the development of interstitial pneumonia accompanied by an Acute Respiratory Distress Syndrome (ARDS), often requiring ventilatory support therapy in Intensive Care Units (ICUs). Current epidemiologic evidence is demonstrating that the COVID-19 prognosis is significantly influenced by its acute complications. Among these, delirium figures as one of the most frequent and severe, especially in the emergency setting, where it shows a significantly negative prognostic impact. In this regard, the aim of our study is to identify clinical severity factors of delirium complicating COVID-19 related-ARDS. We performed a comparative and correlation analysis using demographics, comorbidities, multisystemic and delirium severity scores and anti-delirium therapy in two cohorts of ARDS patients with delirium, respectively, due to COVID-19 (*n* = 40) or other medical conditions (*n* = 39). Our results indicate that delirium in COVID-19-related ARDS is more severe since its onset despite a relatively less severe systemic condition at the point of ICU admission and required higher dosages of antipsychotic and non-benzodiazepinic sedative therapy respect to non-COVID patients. Finally, the correlation analysis showed a direct association between the male gender and maximum dosage of anti-delirium medications needed within the COVID-19 group, which was taken as a surrogate of delirium severity. Overall, our results seem to indicate that pathogenetic factors specifically associated to severe COVID-19 are responsible for the high severity of delirium, paving the way for future research focused on the mechanisms of the cognitive alterations associated with COVID-19.

## Introduction

Coronavirus disease 19 (COVID-19) is an acute illness caused by severe acute respiratory syndrome coronavirus 2 (SARS-CoV-2) (1). COVID-19 constitutes an extraordinary challenge for healthcare systems, due to the overwhelming number of patients requiring hospitalization and simultaneous access to intensive treatments (2). Lombardy was the epicenter of the first outbreak of COVID-19 in a western country, which prompted an unprecedented quick reorganization of the entire healthcare system (3, 4). In this first wave of the pandemic, Intensive Care Units (ICUs) admission rates increase was unprecedent (5) and prompted a quick reorganization of the entire healthcare system (4).

SARS-CoV-2 belongs to the same family of Respiratory Syndrome Coronavirus 1 (SARS-CoV-1) and Middle East Respiratory Syndrome Coronavirus (MERS-CoV), which share similar mechanisms of invasion of the host tissues (6). Noteworthy, coronaviruses are known to cause various neurological manifestations due to their ability to target neurons (i.e., SARS-CoV-1; MERS-CoV; murine hepatitis virus) (7, 8).

Also for SARS-CoV-2, a neuroinvasive potential has been suggested as a possible pathogenetic mechanism responsible for the neurological and psychiatric manifestations often occurring in COVID-19 (9).

The most common neurological manifestations of COVID-19 are encephalopathy, meningoencephalitis, ischemic stroke, acute necrotizing encephalopathy and Guillain-Barrè syndrome (10). Fotuhi et al. identified three stages of neurological impairment based on the clinical severity and the related degree of immunological damage (i.e., neuroCOVID stages I-III) (11). According to this classification, delirium figures among the most serious symptoms of neuroCOVID stage III. Delirium is a common psychiatric complication of several medical conditions, with a significant incidence in hospitalized elderly patients affected by infectious diseases, such as COVID-19 (12). It is characterized by an acute, reversible and fluctuating disorder of attention, cognitive state and level of consciousness, frequently associated with behavioral alterations (13). The pathogenesis of delirium seems multifactorial since it might be due to a direct viral invasion of the neurons and to a neurotoxic effect of the increased cytokine levels often observed in the most severe forms of the disease (14). In critically ill patients, delirium is associated with a higher mortality independently from the severity of the main underlying disease and of the preexisting individual risk factors (12). Studies on large cohorts of ICUs patients have reported delirium to be highly prevalent and prolonged (15) with patients also showing persistent neuropsychological dysfunctions even after discharge (16). However, delirium is highly prevalent in mechanically ventilated patients (17), even though delirium rate in ICU seems to be declining in recent years (18). Thus, whether its prevalence and/or severity is higher in COVID-19 patients is debatable and to date has not been demonstrated.

The aim of this study is to characterize delirium in critically ill patients in the ICU, in terms of risk factors, prevalence, severity and response to interventions. In particular, we hypothesize that delirium associated with Acute Respiratory Distress Syndrome (ARDS) is more frequent and/or severe in COVID-19 patients compared to non-COVID-19 patients in emergency settings and that premorbid and or disease-related risk factors may exist in delirium complicating COVID-19. To test this hypothesis, we used data from a pre-pandemic cohort of non-COVID-19 related ARDS patients from the same ICU as controls.

## Methods

### Participants

We conducted a retrospective observational study on two samples of ICUs patients with delirium complicating an ARDS, respectively, due to COVID-19 (COV+/DEL+ group) or to other medical conditions (COV–/DEL+ group).

Inclusion criteria were hospitalization in ICUs of the IRCSS Ca' Granda, Ospedale Maggiore Policlinico in Milan, Italy affected by COVID-19 (first group) and not affected by COVID-19 (second group).

**Figure 1:**
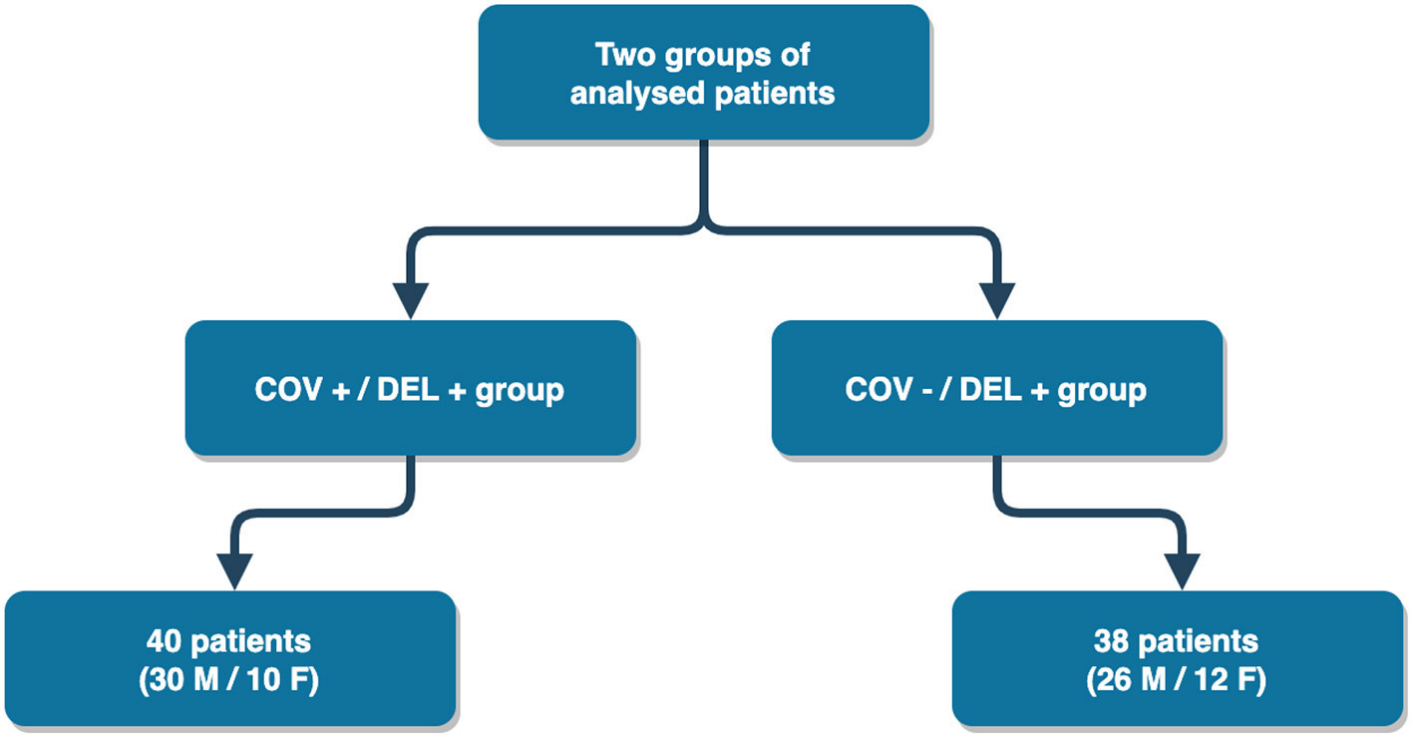
Flowchart depicting the selection process of the patients.

We retrospectively reviewed the electronic health records of a cohort of critically ill adult patients who developed delirium while admitted to the ICUs of the IRCSS Ca' Granda, Ospedale Maggiore Policlinico in Milan, Italy, which is a tertiary referral center for the treatment of patients with acute respiratory failure. The sample included two groups of delirium-complicated patients affected by ARDS due to COVID-19 (COV+/DEL+) or to other diseases (COV–DEL+). Both groups were expected to exhibit symptoms consistent with ARDS and delirium. For the second group (COVID – / DEL +), patients were evaluated before the first case of SARS-CoV-2 diagnosed in China (December 2019). The outline of the two groups is shown in Figure 1, and the descriptions examine the study criteria.

In both groups, ARDS was diagnosed according to Berlin definition criteria (19) and the diagnosis of delirium was made following the DSM-5 criteria (20) and confirmed by the clinical consensus of two psychiatrists. The diagnosis of COVID-19 was based on guidelines issued by the World Health Organization (21) and SARS-CoV-2 infection was confirmed by RT-PCR of upper nasopharyngeal swabs and/or lower respiratory tract aspirates.

The presence of inclusion criteria and the adherence to diagnostic criteria for delirium and ARDS were confirmed by two expert medical researchers of the Psychiatry Unit and two expert intensivists of Ospedale Maggiore Policlinico. At the time of the study, all patients had not been vaccinated since the vaccine against the SARS-CoV-2 virus did not yet exist. The study was approved by the Ethics Committee “Milan Area 2” (trial number: 1732).

### Clinical Assessment and Data Collection

For each patient, the following data were collected retrospectively from the digitalized data records of the Ospedale Maggiore Policlinico Intensive Care Unit:

- demographics, including age, sex and ethnicity
- past medical history
- history of psychiatric disorders
- Sequential Organ Failure Assessment (SOFA) score, that describes the presence and severity of organ failures (respiratory, cardiovascular, renal, neurological, hepatic, and hematological) and is used for the prognostic stratification of ICUs patients (22)
- severity of delirium, assessed using the short form of the Confusion Assessment Method—Severity (CAM-S) (23).
- treatment of delirium (drugs used, maximum dosage administered and duration)

These data were reported in an electronic case report form with the Excel format, in compliance with current privacy regulations. Each participant, at the time of enrollment, was assigned a unique code and data were anonymized: only local investigators could trace the identity of enrolled subjects enrolled. The database was password-protected and accessible only to study personnel designated by the principal investigator.

### Statistical Analysis

A database was created specifically for the management and collection of data using Microsoft Excel, stored in anonymous digital form on the servers of the Policlinico Hospital of Milan. Julia language was used for data preprocessing, analysis, and implementation of the plots and R for basic statistical analyses. We proceeded with a primary consistency check in the data encoding, correcting any input errors with consequent uniformity of the data. Given the non-normal distribution of the variables collected, we performed averages comparisons between the “COVID +; Delirium +”; and “COVID –; Delirium +,” by means of non-parametric statistics. For the between-groups analysis, we selected Mann-Whitney U test for the median comparison of continuous variables and χ^2^ test for testing difference in co-occurrences for dichotomic and categorical variables. Since some anti-delirium drugs (i.e., thioridazine, clonazepam, promazine and olanzapine) were administered to less than the 50% of patients in each group (Table 1), they have not been included in the analysis pipeline. Further, to verify the statistic relationship between premorbid individual factors, systemic involvement and delirium severity within each diagnostic group (COV+ and COV– ARDS complicated by delirium), we performed correlation analyses between three set of variables: (a) baseline variables (demographics and comorbidities); (b) systemic and delirium severity (CAM-S at delirium onset, SOFA total score and single component values at ICU admission) and (c) highest dose needed of anti-delirium therapies administered, considered as surrogate markers of delirium's course severity. For this purpose, we performed Mann–Whitney-*U* tests to correlate binomial categorical with continuous variables, χ^2^ test to analyze differences in joint distributions between categorical variables, and Spearman's rho to tests for exploring the correlations between continuous variables. Finally, the direction (positive/negative) of the significant correlations was inferred from the Spearman's rho for the correlations between continuous variables or between monotonic categorical and binomial variables as well as from the comparison of medians in the correlations between continuous and binomial variables.

**Table 1:** Results from descriptive and comparative analysis carried out in the two groups and including: (a) demographics; (b) presence of comorbidities (hypertension and psychiatric disorders) at ICUs hospitalization, SOFA and CAM-S scores, respectively indicating the systemic severity at ICUs admission and the delirium severity at the onset, and (c) anti-delirium drugs dosage (mg) used in the two groups of patients.

**Variables**	**COV–/DEL+ group**	**COV+/DEL+ group**	**Comparative analysis results**
**a) Demographics**			
Age (years, mean ± std)	Mean: 56.76 ± 11.95 missing: 0 median: 57.5 IQR: 14.0	58.45 ± 8.78 missing: 0 median: 60.0 IQR: 13.0	*U* = 695.0 *p*-value = 0.259
Sex (female = 0, male = 1)	0 → 12 (31.6%) 1 → 26 (68.4%) missing: 0	0 → 10 (25.0%) 1 → 30 (75.0%) missing: 0	χ^2^ = 0.4166 *p*-value = 0.5187 Odds Ratio = 1.3846 Relative Risk = 1.0962
Ethnicity	Hispanic → 1 (2.6%) Asian → 1 (2.6%) African → 4 (10.5%) Caucasian → 32 (84.2%) missing: 0	African → 4 (10.0%) Caucasian → 36 (90.0%) missing: 0	Not tested
**b) Comorbidities and clinical severity scores**			
General comorbidities (*n* = 0, 1–2, >2)	0 → 11 (28.9%) 1–2 → 21 (55.3%) >2 → 6 (15.8%) missing: 0	0 → 15 (37.5%) 1–2 → 17 (42.5%) >2 → 8 (20.0%) missing: 0	χ^2^ = 1.272 *p*-value = 0.529 Odds Ratio = 1.684 Relative Risk = 1.235
Hypertension (Yes/No)	No → 25 (65.8%) Yes → 13 (34.2%) missing: 0	No → 24 (60.0%) Yes → 16 (40.0%) missing: 0	χ^2^ = 0.2796 *p*-value = 0.5969 Odds ratio = 1.2820 Relative risk = 1.1692
Psychiatric comorbidities (Yes/No)	No → 32 (84.2%) Yes → 6 (15.8%) missing: 0	No → 38 (95.0%) Yes → 2 (5.0%) missing: 0	Not tested
SOFA score at ICUs hospitalization (mean ± std)	6.47 ± 2.24 missing: 0 median: 7.0 IQR: 3.75	4.63 ± 2.08 missing: 0 median: 4.0 IQR: 3.0	*U* = 398.0 *p*-value < 0.001
P/F (mmHg) at ICU hospitalization (mean ± std)	136.37 ± 65.45 missing: 0 median: 122.500 IQR: 63.500	141.05 ± 52.70 missing: 2 median: 140.500 IQR: 82.000	*U* = 654.0 *p-*value = 0.242
Platelets (×10^3^/ μL) at ICU hospitalization (mean ± std)	224.74 ± 150.86 missing: 0 median: 211.000 IQR: 155.000	280.75 ± 129.33 missing: 0 median: 264.500 IQR: 142.500	*U* = 545.5 *p*-value = 0.016
Serum creatinine (mg/dl) at ICU hospitalization (mean ± std)	1.33 ± 1.00 missing: 0 median: 0.900 IQR: 0.800	1.07 ± 0.74 missing: 0 median: 0.850 IQR: 0.425	*U* = 654.5 *p*-value = 0.146
Total bilirubin (mg/dl) at ICU hospitalization (mean ± std)	0.74 ± 0.68 missing: 0 median: 0.500 IQR: 0.600	1.04 ± 1.08 missing: 0 median: 0.600 IQR: 0.900	*U* = 582.0 *p*-value = 0.037
Mean arterial pressure OR administration of vasoactive agents required at ICU hospitalization (Cardioscore)	0 → 8 (21.1%) 1 → 5 (13.2%) 2 → 1 (2.6%) 3 → 6 (15.8%) 4 → 18 (47.4%) missing: 0	0 → 28 (70.0%) 1 → 2 (5.0%) 2 → 1 (2.5%) 3 → 6 (15.0%) 4 → 3 (7.5%) missing: 0	*U* = 335.5 *p*-value < 0.001
CAM-S score at delirium onset (mean ± std)	3.37 ± 0.97 missing: 0	3.87 ± 0.80 missing: 1	U = 516.5 p-value = 0.007
**c) Anti-delirium drugs**			
HAL dosage (mg) (mean ± std)	2.76 ± 1.90 median: 2.0 IQR: 2.0 missing: 0	2.45 ± 2.09 median: 2.0 IQR: 3.25 missing: 0	*U* = 692.0 *p*-value = 0.245
QUE dosage (mg) (mean ± std)	89.47 ± 146.19 median: 0.0 IQR: 100.0 missing: 0	161.25 ± 129.34 median: 150.0 IQR: 300.0 missing: 0	*U* = 492.5 *p*-value = 0.003
ALP dosage (mg) (mean ± std)	0.70 ± 0.60 median: 0.75 IQR: 1.375 missing: 0	0.87 ± 0.63 median: 1.0 IQR: 1.5 missing: 1	*U* = 619.5 *p*-value = 0.101
HYD dosage (mg) (mean ± std)	232.89 ± 227.29 median: 200.0 IQR: 300.0 missing: 0	476.32 ± 225.93 median: 600.0 IQR: 275.0 missing: 2	*U* = 344.0 *p*-value < 0.001
LOR (administered, Yes/No)	No → 20 (52.6%) Yes → 18 (47.4%) missing: 0	No → 20 (50.0%) Yes → 19 (47.5%) missing: 1	χ^2^ = 0.014 *p*-value = 0.906 Odds ratio = 0.947 Relative risk = 0.972
MID (administered, Yes/No)	No → 13 (34.2%) Yes → 25 (65.8%) missing: 0	0 → 21 (52.5%) 1 → 16 (40.0%) missing: 3	χ^2^ = 3.845 *p*-value = 0.050 Odds ratio = 2.524 Relative risk = 1.521
TZD (administered, Yes/No)	No → 35 (92.1%) Yes → 3 (7.9%) missing: 0	No → 33 (82.5%) Yes → 6 (15%) missing: 1	Not tested
CLN (administered, Yes/No)	No → 38 (100.0%) Yes → 0 (0%) missing: 0	No → 29 (72.5%) Yes → 10 (25%) missing: 1	Not tested
PMZ (administered, Yes/No)	No → 26 (68.4%) Yes → 12 (31.6%) missing: 0	No → 33 (82.5%) Yes → 6 (15%) missing: 1	Not tested
OLA (administered, Yes/No)	No → 30 (78.9%) Yes → 7 (18.4%) missing: 1	No → 36 (90.0%) Yes → 3 (7.5%) missing: 1	Not tested

*Comparative analysis results are reported as Mann–Whitney U for categorical variables and χ^2^, Odds ratio and Relative risk for numerical variables. For all the comparisons, significance was defined by a p-value < 0.05.*

*Cauc, Caucasian; Afr, African; Asia, Asiatic; Hisp, Hispanic; std, standard deviation, SOFA, Sepsis-related Organ Failure Assessment; ICUs, Intensive Care Unit; CAM-S, Confusion Assessment Method″Short Version; P/F, PaO_2_ to fractional inspired oxygen (FiO_2_) ratio; HAL, haloperidol; QUE, quetiapine; ALP, alprazolam; TZD, thioridazine; HYD, hydroxyzine; LOR, lorazepam; MID, midazolam; CLN, clonazepam; PMZ, promazine; OLA, olanzapine*.

## Results

The sample included two groups of patients, matched for age and sex: (a) the COV+/DEL+ group included patients hospitalized in the ICUs between February 21st 2020 and June 29th 2020 for ARDS due to COVID-19 who required invasive mechanical ventilation and developed delirium during the ICUs stay; (b) the COV-/DEL+ group included patients hospitalized between January 2014 and December 2019 in the same ICUs for ARDS related to different medical conditions requiring invasive mechanical ventilation support and complicated by delirium.

The COV+/DEL+ group was made of 40 patients (30 males and 10 females) with a mean age of 58.45 ± 8.78 years, whilst the COV–/DEL+ group included 38 patients (26 males and 12 females), with a mean age of 56.76 ± 11.95 years. Descriptive and comparative analysis' results of the two study groups are reported in Table 1.

**Figure 2:**
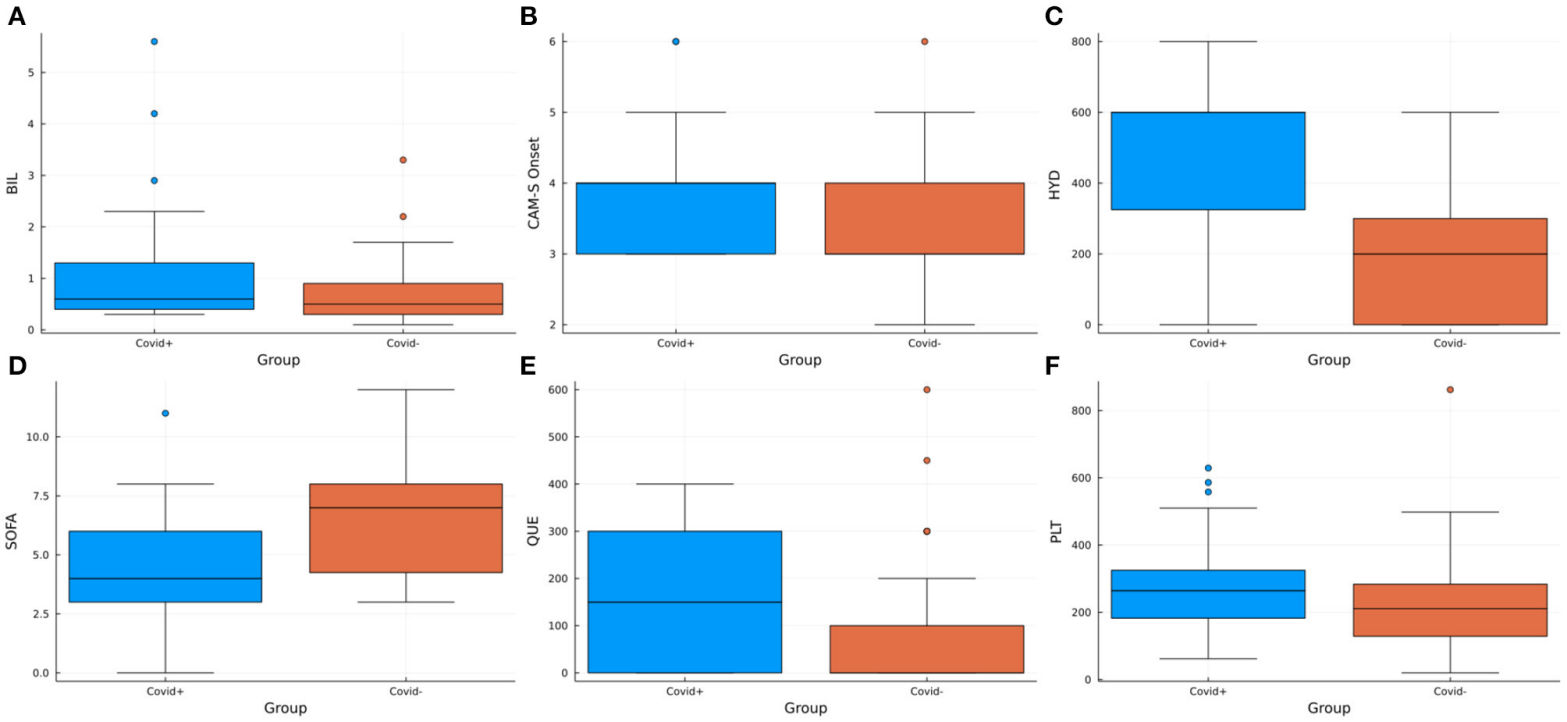
Box plots showing the distribution of the variables resulted significantly different in the comparative analysis between the COV+/DEL+ and COV–/DEL+ groups. BIL, Bilirubin; CAM-S, Confusion Assessment Method—Short Version; HYD, hydroxyzine; PLT, platelets count; QUE, quetiapine; SOFA, Sepsis-related Organ Failure Assessment.

In the comparative analysis, the COV+/DEL+ group presented significantly higher CAM-S scores at delirium onset (*U* = 516.5, *p*-value = 0.007) and significantly lower SOFA scores at ICUs admission (*U* = 398.0, *p*-value < 0.001) compared to COV-/DEL+ patients (Figures 2B,D). Moreover, the group comparison of the SOFA score's individual variables demonstrated significantly higher platelets count (*U* = 545.5, *p*-value = 0.016) and total bilirubin levels (*U* = 582.0, *p*-value = 0.037) in the COV+/DEL+ respect to the COV–/DEL+ group (Figures 2A,F). On the contrary, a lower cardiovascular component score was observed in the COV+/DEL+ (*U* = 335.5 *p*-value < 0.001), showing a predominant administration of high-dose vasopressors (cardiovascular score = 4) in COV–/DEL+ patients (frequency 47.4%) in contrast to predominant normal arterial pressures (cardiovascular score = 0) in the COV+/DEL+ group (frequency 70.0%).

With regards to the drugs used for the treatment of delirium, patients in the COV+/DEL+ group received significantly higher doses of quetiapine (respectively 161.25 mg ± 129.34 vs. 89.47 mg ± 146.19, *U* = 492.5, *p*-value = 0.003) and hydroxyzine (respectively 476.32 mg ± 225.93 vs. 232.89 mg ± 227.29, *U* = 344.0, *p*-value < 0.001) than the COV–/DEL+ group (Figures 2C,E). Also, a more extensive use of midazolam was observed in the COV+ respect to the COV– patients (χ^2^ = 3.845, *p*-value = 0.050) (Table 1).

### Within-Group Correlations

**Figure 3:**
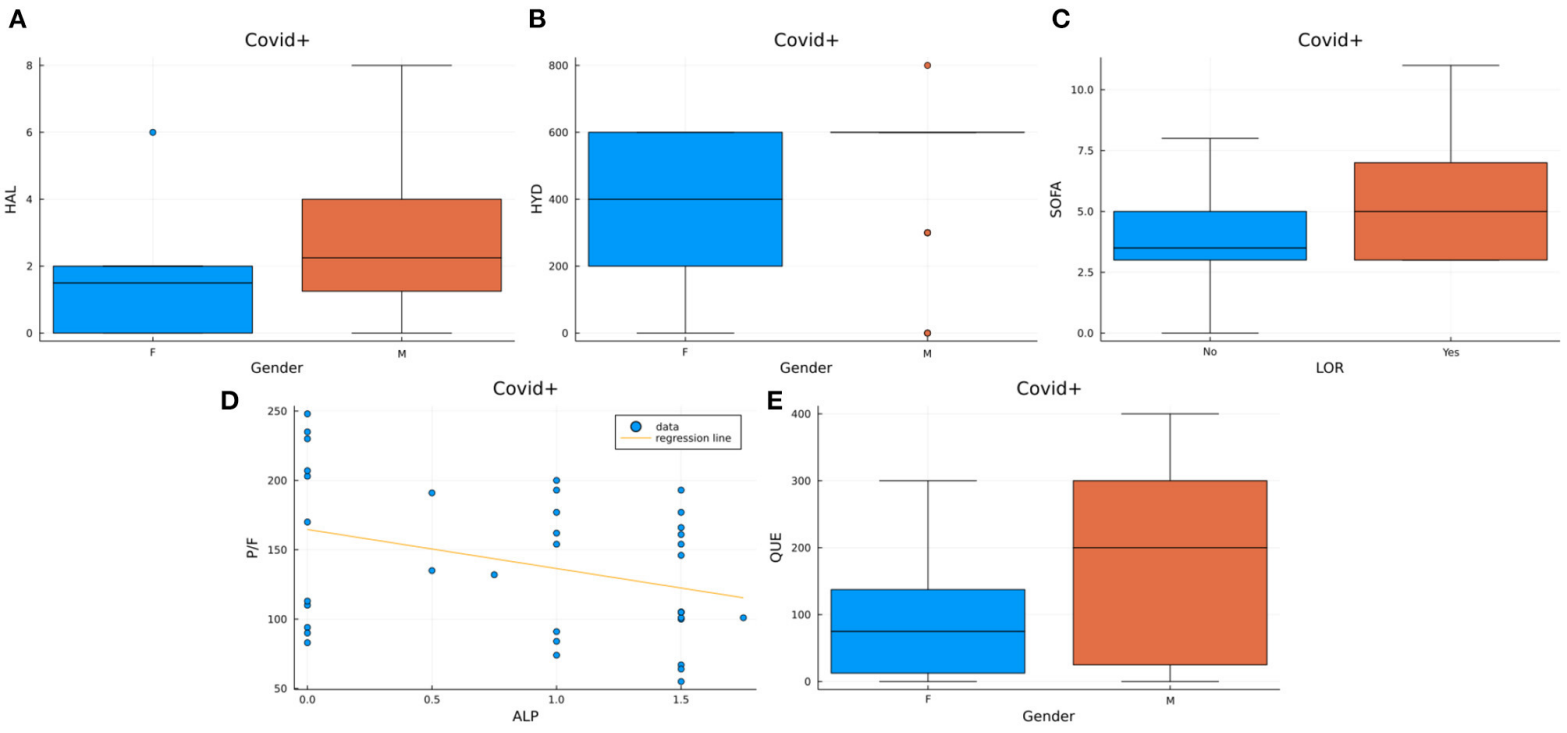
Visualization of the significant results from the correlations in the COV+/DEL+ group. Box plots represent the distribution of haloperidol (Figure 2A), quetiapine (Figure 2E) and hydroxyzine (Figure 2B) maximum dosages for each sex, SOFA scores in subjects assuming LOR vs. subjects not assuming LOR (Figure 2C). The scatter plot with regression line (Figure 2D) represents the distribution of P/F values vs. the maximum dose of alprazolam used. HAL, Haloperidol; HYD, Hydroxyzine; SOFA, Sepsis-related Organ Failure Assessment; P/F, PaO2/FIO2; QUE, Quetiapine; LOR, Lorazepam; ALP Alprazolam.

**Figure 4:**
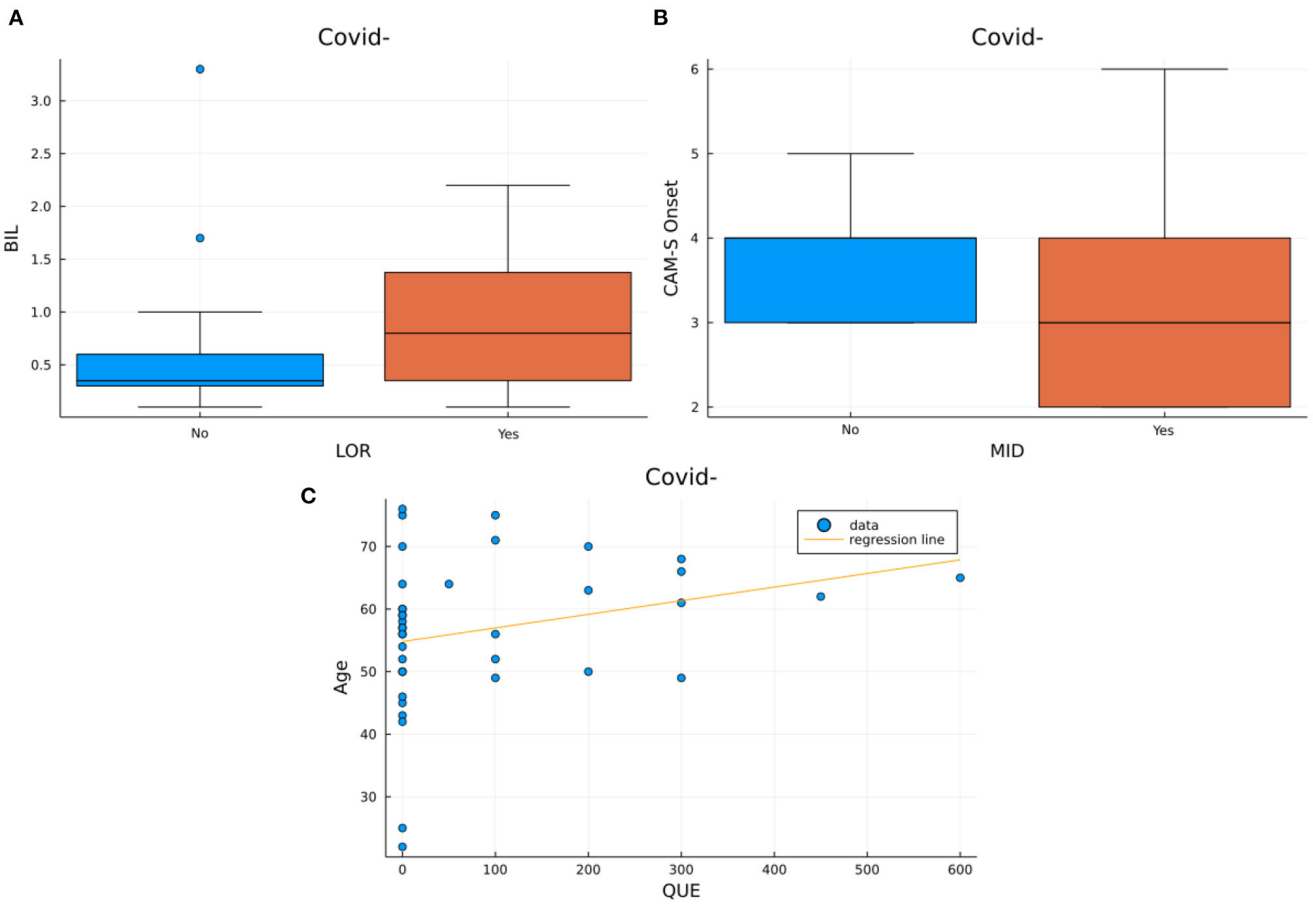
Visualization of the significant results from the correlations in the COV–/DEL+ group. Box plots represent the bilirubin levels distributions in subjects assuming LOR vs. subjects not assuming LOR (Figure 3A) and the distribution of different CAM-S scores at the onset of delirium in subjects assuming MID and in subjects not assuming MID (Figure 3B). The scatter plot with regression line (Figure 2D) represent age vs. maximum dose of quetiapine used.

The correlation analysis performed within each patients' group demonstrated two different patterns of significant correlations in the COV+ and COV– delirium groups (Table 2). In the COV+/DEL+ group we observed a correlation between male sex and the dose of haloperidol (*U* = 92.500, *p*-value = 0.035), quetiapine (*U* = 97.500, *p*-value = 0.047), hydroxyzine (*U* = 83.000, *p*-value = 0.025) and lorazepam (χ^2^ = 4.439, *p*-value = 0.035) administered. Also, positive correlations have been found between the dose of lorazepam and SOFA scores (*U* = 130.500, *p*-value = 0.044) as well as between the quetiapine and hydroxyzine dose and the SOFA's cardiovascular component (respectively *U* = 5.000, *p*-value = 0.028 and *U* = 9.000, *p*-value = 0.038). On the contrary, a negative correlation between the dose of alprazolam and the PaO_2_ to FiO_2_ ratio was demonstrated (ρ = −0.325, *p*-value = 0.049) (Figure 3). None of these correlations were observed in the COV–/DEL+ group, in which, instead, we observed a positive correlation between age and quetiapine dose (ρ = 0.327, *p*-value = 0.045) and between total bilirubin at ICU admission and lorazepam dose (*U* = 120.00, *p*-value = 0.041) as well as a negative correlation between midazolam administration and CAM-S at delirium onset (*U* = 104.00, *p*-value = 0.038) (Figure 4).

**Table 2:** Significant results obtained from the within-group correlation analyses between (a) baseline variables: demographics and comorbidities; (b) systemic and delirium severity: CAM-S at delirium onset, SOFA total score and single components at ICU admission and (c) highest dose needed of anti-delirium therapies administered.

**Coupled variables**	**Mann-Whitney-U**	**Chi-square**	**Spearman-rho**	***p*-value**	**Correlation sign**
**1) COV–/DEL+ group**					
QUE ~ Age			0.327	0.045	Positive
LOR ~ BIL	120.500			0.041	Positive
MID ~ CAM-S Onset	104.000			0.030	Negative
**2) COV+/DEL+ group**					
LOR ~ Sex		4.439		0.035	Positive
HAL ~ Sex	92.500			0.035	Positive
QUE ~ Sex	97.500			0.047	Positive
HYD ~ Sex	83.000			0.025	Positive
LOR ~ SOFA	130.500			0.044	Positive
ALP ~ P/F			−0.325	0.049	Negative

*For each couple of variables, the direction of the correlation (positive/negative) is indicated. For correlations with the “Sex” variable, a positive and negative direction referred to an increased frequency of males or females*.

*SOFA, Sepsis-related Organ Failure Assessment; ICUs, Intensive Care Unit; BIL, Total bilirubin at ICU admission; P/F, PaO_2_ to fractional inspired oxygen (FiO_2_) ratio; CAM-S onset, Confusion Assessment Method—Short Version score at delirium onset; HAL, haloperidol; QUE, quetiapine; ALP, alprazolam; TZD, thioridazine; HYD, hydroxyzine; LOR, lorazepam; MID, midazolam*.

## Discussion

In this retrospective observational study involving two cohorts of patients with ARDS due to COVID-19 or to other medical conditions who developed delirium during ICUs stay, we primarily aimed at describing demographics, clinical features, and anti-delirium treatments. Furthermore, we performed a comparative analysis between the COV+/DEL+ and COV–/DEL+ groups as well as a set of within-group correlation analyses to explore the relationship between baseline variables, systemic involvement and delirium severity, as indicated by the CAM-S score at the onset and by the highest dose of anti-delirium therapies administered, used as surrogate markers of delirium course severity.

Our comparative analysis demonstrated a significant difference between the two groups in terms of clinical severity scores. In particular, the concomitant observation of lower mean SOFA score and higher mean CAM-S scores in the COVID-19 respect to the non-COVID-19 group seem to suggest that despite COV–/DEL+ patients had a more severe multisystemic clinical impairment (as demonstrated by higher SOFA score) at ICUs admission delirium complicating COVID-related ARDS was more severe since its onset. By comparing each single components forming the SOFA score, we observed that higher mean bilirubin levels and blood platelets count, both within normal limits, were found in the COVID-19 respect to the non-COVID-19 patients. On the contrary, a higher cardiovascular score was observed in the non-COVID-19 group, in which most patients required a treatment with high dose vasopressors (47% with cardiovascular score = 4), respect to most COVID-19 patients, which showed, instead, a Mean Arterial Pressure (MAP) ≥70 mmHg (70% with cardiovascular score = 0) at ICU admission. Notably, this latter result is in line with previous evidence having demonstrated a less extensive use of vasopressors at ICU admission and a later vasopressors commencement over the ICU stay in COVID-19 pneumonia respect to non-COVID community acquired pneumonia (24). Also, the higher dosage of quetiapine and hydroxyzine administered in the COVID-19 group seem to confirm the higher severity of delirium course in COVID-19 (25). From the correlation analyses performed within both diagnostic groups, the most consistent result was a significant positive correlation between male sex and the highest dose of almost all anti-delirium treatments used, including haloperidol, quetiapine, hydroxyzine and lorazepam, in the COVID-19 but not in the non-COVID-19 patients. These results are in line with a multicentric observational study reporting a lower impact of delirium on the illness outcome in COVID-19 female patients, possibly addressing for sex-specific pathophysiology conditioning delirium severity in COVID-19 (15). Conversely, the only negative correlation found in the COVID-19 group was between the PaO_2_ to FiO_2_ ratio and alprazolam maximum dose, consistently with a limited use of benzodiazepine in worst respiratory conditions. Thus, the contrast between the lower systemic severity of ARDS and the higher severity of delirium in patients with COVID-19 observed in our analysis could be explained by the peculiar neuro-invasive capacity of SARS-CoV-2 and its association with cognitive impairment, frequently evoked by the most recent literature (26, 27). Moreover, the results of our correlation analyses seem to suggest that male sex might be a major factor associated to a worst course of delirium complicating COVID-19. To verify these hypotheses, future studies should integrate clinical, neuroimaging and neurophysiologic data, as well as laboratory data (including markers of brain invasion, i.e., SARS-CoV-2 RNA in CSF), and findings from post-mortem series of COVID victims affected by delirium, also addressing possible sex-related differences in delirium pathophysiology.

Finally, current literature is increasingly focusing on the long-term effects of COVID-19 on patients recovered from the acute phase of the disease, leading to the definition of a “post-COVID syndrome,” characterized by long-term heterogenous and chronic symptoms. This syndrome seems to be more frequent in subjects that suffered from severe ARDS requiring invasive respiratory support during the acute phase (28). Interestingly, among the most frequent manifestation there are cognitive and psychiatric symptoms, such as sleep impairment, which are commonly observed in patients with delirium (29, 30). Thus, the impact of delirium in favoring the persistence of long-term sequelae after severe COVID-19 should be addressed in longitudinal studies.

Regarding the composition of pharmacologic delirium therapy, patients from the COV–/DEL+ group were treated with higher doses of the benzodiazepine midazolam compared to the COV+/DEL+ group (0.66 mg ± 0.48 vs. 0.43 mg ± 0.50 *p* = 0.05). Considering that our cohort of patients had been hospitalized during the early phase of the pandemic, characterized by a scarcity of clinical guidelines for the treatment of the COVID-19-associated delirium, these data could reflect the tendency of the ICUs clinicians to privilege a therapy with lower doses of benzodiazepines, probably based mainly on clinical observation. Accordingly, a recent longitudinal study has demonstrated that treatment with benzodiazepines is one of the most important risk factors for delirium in ICUs-hospitalized COVID-19, discouraging the benzodiazepines treatment in these patients (15).

## Conclusions, Limits, and Future Perspectives

After several months from the pandemic outbreak, medical literature has well-demonstrated how the onset of delirium can worsen the course and prognosis of COVID-19 patients, especially in the ICUs setting (15, 31). However, the reasons for the high prevalence of this complication in severe COVID-19 remains unclear, and an efficacy preventive and/or therapeutical strategy is an urgent clinical need in order to reduce the ICUs mortality of COVID-19 patients (15). To our knowledge, this is the first observational study comparing ICUs COV+/DEL+ and COV–/DEL+ patients in terms of demographic, clinical and therapeutic features, with the aim to identify factors related to delirium severity in COVID-19 patients. Our descriptive and comparative analyses indicate that, compared to non-COVID delirium, delirium in COVID-19-related ARDS was more severe from its onset, despite a relatively less severe systemic impairment at ICUs admission, and required higher dosages of antipsychotic and sedative therapy (quetiapine and hydroxyzine, respectively). Conversely, in line with the most recent literature, lower dosages of benzodiazepines (i.e., midazolam) were used in our cohorts of COVID-19 compared to non-COVID patients, probably as a consequence of the clinical observation of their lack of efficacy. Finally, a positive correlation was found between male sex and the maximum dosage of anti-delirium therapies in the COVID-19, but not in the COV–/DEL+ group. This data seems to indicate male sex as a relevant severity factor of COVID-related delirium, addressing for a possible sex-specific pathophysiology of this complication. In conclusion, our results may provide preliminary indications in the prevention and management of delirium in ICU COVID-19 patients, possibly supporting the application of early preventive strategies even in patients with a mild systemic impairment. Moreover, the possibility of an increased risk of severe delirium in male patients should be considered early in the clinical management of ICU COVID-19 patients.

Some limitations of our study must be addressed. Firstly, the low sample size and the retrospective design limited the statistical power of our results and the predictive potential of our analysis. Moreover, the overload of Italian Hospitals during the first phase of the pandemic limited the possibility to better characterize COVID patients with laboratory, neuroimaging and electrophysiologic exams in a long-term follow-up, in order to screen for residual cognitive symptoms and outcome at more distant time points. Thirdly, a longitudinal, serial assessment with CAM-S could have permitted to better define the course of delirium, helping to identify prognostic factors.

Therefore, future longitudinal and multicentric studies on large cohorts of COVID-19 patients are required to identify diagnostic prognostic and indices of delirium in order to minimize the clinical burden associated with this severe complication.

## Data Availability Statement

The raw data supporting the conclusions of this article will be made available by the authors, without undue reservation.

## Ethics Statement

The studies involving human participants were reviewed and approved by the Ethics Committee of the Fondazione IRCCS Ca 'Granda Ospedale Maggiore Policlinico, Milan, protocol number 1732. Written informed consent from the patients/participants was not required to participate in this study in accordance with the national legislation and the institutional requirements.

## Author Contributions

PB and GG designed this particular study and coordinated data management. DM, PE, VC, FC, and NT prepared the first version of the manuscript. GD revised the first draft of the manuscript. NT carried out the data analysis. PB, GG, and VC coordinated subject recruitment and data collection. DM, PE, EC, FC, AB, SD, MC, MS, and GC were involved in patient enrolment and assessment. All authors revised and approved the final version of the manuscript.

## Funding

This work has been supported by Fondazione Cariplo (PI: Paolo Brambilla, ID 1854925, Cariplo GRANT ID: 2020-1366).

## Conflict of Interest

The authors declare that the research was conducted in the absence of any commercial or financial relationships that could be construed as a potential conflict of interest.

## Publisher's Note

All claims expressed in this article are solely those of the authors and do not necessarily represent those of their affiliated organizations, or those of the publisher, the editors and the reviewers. Any product that may be evaluated in this article, or claim that may be made by its manufacturer, is not guaranteed or endorsed by the publisher.
