# Supplementary material for: Factors Associated With Severity of Delirium Complicating COVID-19 in Intensive Care Units
Source: Front Neurol. 2022 Mar 24;13:774953. doi: 10.3389/fneur.2022.774953 (PMC8987982; doi:10.3389/fneur.2022.774953)
Supplement: Supplementary file 1 [file Data_Sheet_1.docx]

**Table S1.** Results of the correlation analysis in the COV-/DEL+ group

| **Variables** | **Mann-Whitney U** | **Chi square** | **Spearman rho** | **p-value** |
| --- | --- | --- | --- | --- |
| LOR ~ Sex |  | 0.049 |  | 0.825 |
| LOR ~ Hypertension |  | 0.012 |  | 0.914 |
| LOR ~ Psychiatric comorbidities |  | 0.563 |  | 0.453 |
| MID ~ Sex |  | 0.006 |  | 0.938 |
| MID ~ Hypertension |  | 0.159 |  | 0.690 |
| MID ~ Psychiatric comorbidities |  | 0.974 |  | 0.324 |
| CAM-S Onset ~ Sex | 130.5 |  |  | 0.204 |
| CAM-S Onset ~ Hypertension | 114.0 |  |  | 0.060 |
| CAM-S Onset ~ Psychiatric comorbidities | 65.5 |  |  | 0.103 |
| HAL ~ Sex | 153.5 |  |  | 0.474 |
| HAL ~ Hypertension | 142.5 |  |  | 0.268 |
| HAL ~ Psychiatric comorbidities | 84.0 |  |  | 0.318 |
| QUE ~ Sex | 113.5 |  |  | 0.067 |
| QUE ~ Hypertension | 136.0 |  |  | 0.182 |
| QUE ~ Psychiatric comorbidities | 92.5 |  |  | 0.446 |
| ALP ~ Sex | 138.5 |  |  | 0.291 |
| ALP ~ Hypertension | 131.5 |  |  | 0.167 |
| ALP ~ Psychiatric comorbidities | 83.5 |  |  | 0.311 |
| HYD ~ Sex | 129.0 |  |  | 0.196 |
| HYD ~ Hypertension | 136.5 |  |  | 0.210 |
| HYD ~ Psychiatric comorbidities | 76.0 |  |  | 0.211 |
| Age ~ LOR | 145.0 |  |  | 0.156 |
| Age ~ MID | 130.0 |  |  | 0.162 |
| SOFA ~ LOR | 168.5 |  |  | 0.372 |
| SOFA ~ MID | 152.5 |  |  | 0.384 |
| P/F ~ LOR | 154.5 |  |  | 0.232 |
| P/F ~ MID | 158.0 |  |  | 0.451 |
| PLT ~ LOR | 152.5 |  |  | 0.215 |
| PLT ~ MID | 136.5 |  |  | 0.216 |
| CREA ~ LOR | 174.0 |  |  | 0.436 |
| CREA ~ MID | 159.5 |  |  | 0.469 |
| BIL ~ LOR | 120.5 |  |  | 0.041 |
| BIL ~ MID | 118.0 |  |  | 0.087 |
| CAM-S Onset ~ LOR | 154.5 |  |  | 0.221 |
| CAM-S Onset ~ MID | 104.0 |  |  | 0.030 |
| CAM-S Onset ~ Age |  |  | 0.144 | 0.387 |
| CAM-S Onset ~ SOFA |  |  | 0.279 | 0.090 |
| CAM-S Onset ~ P/F |  |  | -0.287 | 0.081 |
| CAM-S Onset ~ PLT |  |  | 0.246 | 0.137 |
| CAM-S Onset ~ CREA |  |  | 0.070 | 0.676 |
| CAM-S Onset ~ BIL |  |  | -0.206 | 0.215 |
| HAL ~ Age |  |  | 0.082 | 0.623 |
| HAL ~ SOFA |  |  | 0.303 | 0.065 |
| HAL ~ P/F |  |  | -0.232 | 0.161 |
| HAL ~ PLT |  |  | -0.220 | 0.185 |
| HAL ~ CREA |  |  | 0.060 | 0.721 |
| HAL ~ BIL |  |  | -0.020 | 0.903 |
| HAL ~ CAM-S Onset |  |  | 0.106 | 0.526 |
| QUE ~ Age |  |  | 0.327 | 0.045 |
| QUE ~ SOFA |  |  | 0.058 | 0.730 |
| QUE ~ P/F |  |  | 0.022 | 0.897 |
| QUE ~ PLT |  |  | 0.043 | 0.796 |
| QUE ~ CREA |  |  | 0.043 | 0.797 |
| QUE ~ BIL |  |  | 0.191 | 0.250 |
| QUE ~ CAM-S Onset |  |  | -0.055 | 0.743 |
| ALP ~ Age |  |  | 0.116 | 0.487 |
| ALP ~ SOFA |  |  | 0.045 | 0.790 |
| ALP ~ P/F |  |  | -0.110 | 0.512 |
| ALP ~ PLT |  |  | 0.192 | 0.248 |
| ALP ~ CREA |  |  | -0.168 | 0.313 |
| ALP ~ BIL |  |  | 0.263 | 0.111 |
| ALP ~ CAM-S Onset |  |  | 0.044 | 0.794 |
| HYD ~ Age |  |  | 0.037 | 0.828 |
| HYD ~ SOFA |  |  | -0.135 | 0.419 |
| HYD ~ P/F |  |  | 0.181 | 0.276 |
| HYD ~ PLT |  |  | 0.042 | 0.802 |
| HYD ~ CREA |  |  | -0.061 | 0.714 |
| HYD ~ BIL |  |  | 0.160 | 0.339 |
| HYD ~ CAM-S Onset |  |  | -0.288 | 0.080 |

Note: Cauc, Caucasian; Afr, African; Asia, Asiatic; Hisp, Hispanic; std, standard deviation, SOFA, Sepsis-related Organ Failure Assessment; CAM-S, Confusion Assessment Method – Short Version; P/F, PaO2 to fractional inspired oxygen (FiO2) ratio; PLT, blood platelets count; CREA, serum creatinine; BIL, blood total bilirubin; HAL, haloperidol; QUE, quetiapine; ALP, alprazolam; TZD, thioridazine; HYD, hydroxyzine; LOR, lorazepam; MID, midazolam; CLN, clonazepam; PMZ, promazine; OLA, olanzapine.

**Table S2**. Results of the correlation analysis in the COV+/DEL+ group

| **Variables** | **Mann Whitney U** | **Chi square** | **Spearman rho** | **p-value** |
| --- | --- | --- | --- | --- |
| LOR ~ Sex |  | 4.439 |  | 0.035 |
| LOR ~ Hypertension |  | 0.208 |  | 0.648 |
| LOR ~ Psychiatric comorbidities |  | 0.001 |  | 0.970 |
| MID ~ Sex |  | 0.979 |  | 0.322 |
| MID ~ Hypertension |  | 0.187 |  | 0.666 |
| MID ~ Psychiatric comorbidities |  | 1.611 |  | 0.204 |
| CAM-S Onset ~ Sex | 132.0 |  |  | 0.464 |
| CAM-S Onset ~ Hypertension | 160.5 |  |  | 0.273 |
| CAM-S Onset ~ Psychiatric comorbidities | 27.5 |  |  | 0.264 |
| HAL ~ Sex | 92.5 |  |  | 0.035 |
| HAL ~ Hypertension | 188.5 |  |  | 0.466 |
| HAL ~ Psychiatric comorbidities | 22.5 |  |  | 0.171 |
| QUE ~ Sex | 97.5 |  |  | 0.047 |
| QUE ~ Hypertension | 141.0 |  |  | 0.075 |
| QUE ~ Psychiatric comorbidities | 6.0 |  |  | 0.022 |
| ALP ~ Sex | 137.5 |  |  | 0.407 |
| ALP ~ Hypertension | 136.5 |  |  | 0.098 |
| ALP ~ Psychiatric comorbidities | 34.5 |  |  | 0.447 |
| HYD ~ Sex | 83.0 |  |  | 0.025 |
| HYD ~ Hypertension | 160.5 |  |  | 0.295 |
| HYD ~ Psychiatric comorbidities | 12.5 |  |  | 0.034 |
| Age ~ LOR | 172.0 |  |  | 0.311 |
| Age ~ MID | 115.5 |  |  | 0.055 |
| SOFA ~ LOR | 130.5 |  |  | 0.044 |
| SOFA ~ MID | 143.0 |  |  | 0.219 |
| P/F ~ LOR | 170.5 |  |  | 0.500 |
| P/F ~ MID | 119.5 |  |  | 0.145 |
| PLT ~ LOR | 133.0 |  |  | 0.056 |
| PLT ~ MID | 157.0 |  |  | 0.374 |
| CREA ~ LOR | 160.0 |  |  | 0.203 |
| CREA ~ MID | 119.5 |  |  | 0.070 |
| BIL ~ LOR | 168.5 |  |  | 0.276 |
| BIL ~ MID | 121.0 |  |  | 0.075 |
| CAM-S Onset ~ LOR | 133.5 |  |  | 0.069 |
| CAM-S Onset ~ MID | 146.5 |  |  | 0.324 |
| CAM-S Onset ~ Age |  |  | -0.017 | 0.918 |
| CAM-S Onset ~ SOFA |  |  | 0.021 | 0.897 |
| CAM-S Onset ~ P/F |  |  | -0.161 | 0.342 |
| CAM-S Onset ~ PLT |  |  | -0.170 | 0.300 |
| CAM-S Onset ~ CREA |  |  | -0.261 | 0.108 |
| CAM-S Onset ~ BIL |  |  | -0.030 | 0.856 |
| HAL ~ Age |  |  | -0.067 | 0.681 |
| HAL ~ SOFA |  |  | 0.295 | 0.065 |
| HAL ~ P/F |  |  | 0.003 | 0.984 |
| HAL ~ PLT |  |  | -0.008 | 0.959 |
| HAL ~ CREA |  |  | -0.051 | 0.755 |
| HAL ~ BIL |  |  | -0.083 | 0.611 |
| HAL ~ CAM-S Onset |  |  | 0.303 | 0.060 |
| QUE ~ Age |  |  | -0.050 | 0.761 |
| QUE ~ SOFA |  |  | 0.275 | 0.086 |
| QUE ~ P/F |  |  | -0.315 | 0.054 |
| QUE ~ PLT |  |  | -0.211 | 0.191 |
| QUE ~ CREA |  |  | 0.119 | 0.463 |
| QUE ~ BIL |  |  | -0.005 | 0.977 |
| QUE ~ CAM-S Onset |  |  | 0.133 | 0.421 |
| ALP ~ Age |  |  | 0.077 | 0.640 |
| ALP ~ SOFA |  |  | -0.121 | 0.463 |
| ALP ~ P/F |  |  | -0.325 | 0.049 |
| ALP ~ PLT |  |  | -0.036 | 0.829 |
| ALP ~ CREA |  |  | 0.030 | 0.854 |
| ALP ~ BIL |  |  | 0.083 | 0.614 |
| ALP ~ CAM-S Onset |  |  | -0.139 | 0.406 |
| HYD ~ Age |  |  | -0.320 | 0.050 |
| HYD ~ SOFA |  |  | -0.061 | 0.716 |
| HYD ~ P/F |  |  | -0.310 | 0.066 |
| HYD ~ PLT |  |  | 0.074 | 0.660 |
| HYD ~ CREA |  |  | 0.079 | 0.639 |
| HYD ~ BIL |  |  | -0.247 | 0.135 |
| HYD ~ CAM-S Onset |  |  | -0.005 | 0.978 |

Note: Cauc, Caucasian; Afr, African; Asia, Asiatic; Hisp, Hispanic; std, standard deviation, SOFA, Sepsis-related Organ Failure Assessment; CAM-S, Confusion Assessment Method – Short Version; P/F, PaO2 to fractional inspired oxygen (FiO2) ratio; PLT, blood platelets count; CREA, serum creatinine; BIL, blood total bilirubin; HAL, haloperidol; QUE, quetiapine; ALP, alprazolam; TZD, thioridazine; HYD, hydroxyzine; LOR, lorazepam; MID, midazolam; CLN, clonazepam; PMZ, promazine; OLA, olanzapine.
